# Prognostic values of hematological biomarkers in nasopharyngeal carcinoma patients treated with intensity-modulated radiotherapy

**DOI:** 10.1007/s00405-018-4956-x

**Published:** 2018-03-27

**Authors:** Lulu Ye, Ronald Wihal Oei, Fangfang Kong, Tingting Xu, Chunying Shen, Xiaoshen Wang, Xiayun He, Lin Kong, Chaosu Hu, Hongmei Ying

**Affiliations:** 1 Department of Radiation Oncology, Fudan University Shanghai Cancer Center, 270 Dongan Road, Shanghai, 200032 People’s Republic of China; 2 Department of Oncology, Shanghai Medical College, Fudan University, 138 Yixueyuan Road, Shanghai, 200032 People’s Republic of China

**Keywords:** Nasopharyngeal carcinoma, Biomarkers, Intensity-modulated radiotherapy, Survival

## Abstract

**Purpose:**

In this study, we evaluated the prognostic values of hematological biomarkers in primary nasopharyngeal carcinoma (NPC) patients receiving definitive intensity-modulated radiotherapy (IMRT).

**Methods:**

There were 427 NPC patients enrolled between January 2010 and March 2013 at Fudan University Shanghai Cancer Center. Pre-treatment absolute neutrophil count (ANC), platelet count (APC), lymphocyte count (ALC), neutrophil-to-lymphocyte ratio (NLR) and platelet-to-lymphocyte ratio (PLR) were collected as prognostic biomarkers. The Kaplan–Meier method and log-rank test were utilized to calculate progression-free survival (PFS) and overall survival (OS). The Cox proportional hazard models were applied to assess variables.

**Results:**

ANC, APC and ALC were declined, while NLR and PLR were elevated significantly after therapy (*P* < 0.001 each). On multivariate analysis, pre-treatment NLR ≥ 2.32 was associated with shortened OS (*P* = 0.048) and PFS (*P* = 0.008), whereas PLR ≥ 123.0 was related with inferior OS (*P* = 0.032), yet it was not correlated with PFS (*P* = 0.161).

**Conclusions:**

High pre-treatment NLR and PLR indicated poor survival in NPC patients treated with IMRT-based therapy. As easily accessible and economically feasible biomarkers, NLR and PLR can be applied into clinical practice, in combination with current TNM staging, to design a more personalized treatment in these patients.

## Introduction

Nasopharyngeal carcinoma (NPC) is one of the high-occurrence cancer among head and neck malignancies with distinguished racial and geographic distributions. NPC is epidemic in Southeast Asia, most particularly in Canton (China), with an incidence of 20–30 in 100,000 persons per year [1, 2]. Since its asymptomatic characteristic in early stage, most patients present with advanced stage at primary diagnosis. Though NPC is a radio-sensitive disease, its prognostic outcomes vary despite even in the same stages, as a consequence, TNM staging-based therapy protocol is insufficient. In the last decade, the research on the prognostic hematological biomarkers in different cancers have gained notable progress. High level of pre-treatment absolute neutrophil count (ANC), platelet count (APC) or low lymphocyte count (ALC) were regarded as negative predictors in various cancers, such as gallbladder [3], ovarian carcinomas [4], etc. In addition, above markers could be combined into ratios including neutrophil-to-lymphocyte ratio (NLR) and platelet-to-lymphocyte ratio (PLR). High NLR and PLR were believed to correlate with inferior outcomes, as reported in digestive system cancers [3], lung [5] and many other types of solid tumors [6].

Since the patients with head and neck squamous cell carcinoma (HNSCC) have been revealed to have a higher level of neutrophils compared with healthy subjects [7], there has been rising interest in the study of hematological biomarkers in HNSCC in the recent years [7, 8], as well as in NPC [1, 2, 9]. On these bases, we supposed that hematological biomarkers may be related to the local–regional relapse and distant metastasis in NPC patients. The purpose of this study was to investigate the prognostic values of these markers.

## Patients and methods

### Patient population

This study was approved by Institutional Review Board of Fudan University Shanghai Cancer Center and run in full accordance with ethical principles set in our institutional policy and the World Medical Association Declaration of Helsinki. Due to retrospective nature of the study, we requested and were granted a waiver of written informed consent.

The eligibility criteria were: (1) patients with age of 16 years old and above; (2) primary diagnosed NPC patients with biopsy-confirmed World Health Organization (WHO) type II or III; (3) no evidence of distant metastasis; (4) treated with definitive intensity modulated radiotherapy (IMRT) at our hospital between January 2010 and March 2013; (5) complete pre- and post-treatment data. The exclusion criteria were: (1) presence of distant metastasis at diagnosis; (2) underwent neck nodal dissection prior to radiotherapy; (3) irradiation to head and neck before or recurrence in nasopharynx; (4) confirmed hematological disorders or even with a single episode of systemic infection before or at diagnosis; (5) Karnofsky Performance Score (KPS) < 70; (6) with follow-up for less than 1 year.

All patients were screened before treatment with complete medical history, physical examination, magnetic resonance imaging (MRI) of nasopharynx and neck, chest computed tomography (CT) scan, abdominal ultrasonography, single-photon emission computed tomography (SPECT) for whole-body bone scan, as well as hematology test, including complete blood count, hepatic and renal function tests. The tumor staging was defined by the 7th edition of the American Joint Committee on Cancer (AJCC) staging system for NPC. The blood samples were collected within 14 days before and after treatment. The hematological biomarkers were measured by a fully automated hematology analyzer Sysmex XT-4000i (Sysmex, Kobe, Japan). A total of 427 patients were enrolled in the final analysis.

### Treatment

All patients were treated with definitive radiotherapy or chemoradiotherapy. TNM staging was the guide of treatment plan. According to the institutional guidelines, radical radiotherapy alone was provided to patients in stage I, concurrent chemoradiotherapy for stage II patients, while concurrent chemoradiotherapy with or without neoadjuvant/adjuvant chemotherapy for stage III or IV patients. The radiotherapy was given in the form of IMRT with six megavoltage photons (6 MV). It was performed in a daily fraction of 2.0–2.2 Gy, 5 days per week for 6–7 weeks. The total radiation dosage was 66 or 70.4 Gy to primary lesion of nasopharynx (66 Gy for T1–T2, 70.4 Gy for T3–T4), 66 or 70.4 Gy to metastatic lymph nodes of the neck, 60 Gy to the regions of high-risk microinvasive lymphatic drainage areas (clinical target volume 1, CTV1) and 54 Gy to low-risk areas (clinical target volume 2, CTV2). Concurrent chemotherapy was cisplatin which dosed at 80 mg/m^2^ every 3 weeks or 40 mg/m^2^ weekly, neoadjuvant or adjuvant chemotherapy consisted of 2–3 cycles of cisplatin-based regimens administered every 3 weeks.

### Follow-up

After the completion of treatment, patients received regular examinations at our outpatient clinics every 3 months during the first 2 years, every 6–9 months in the 3rd to 5th years, and annually thereafter. Salvage treatments such as neck nodal dissection, re-radiotherapy or systemic chemotherapy were provided to patients with confirmed local–regional relapse or distant metastatic event.

The primary endpoint was overall survival (OS) which defined as the duration from the initiation of treatment to death of any cause. The secondary endpoint was progression-free survival (PFS) which measured from the beginning of therapy to local–regional relapse or distant metastasis or all-cause death. For patients who were still alive or with no progressive disease, the latest date of follow-up was recorded.

### Statistical analysis

The Statistical Package for Social Sciences (version 21.0, IBM Corporation, Armonk, NY, USA) was used for statistical analysis. NLR was calculated as ANC divided by ALC, whereas PLR was quantified as APC divided by ALC. Continuous variables were expressed as median with range or mean with standard deviation, and categorical variables as number and percentage. Paired sample t test with Bonferroni correction was applied to compare the variables (including ANC, APC, ALC, NLR, and PLR) at pre- and post-treatment. Kaplan–Meier method was used to assess OS and PFS, and log-rank test to compare the survival rates of two samples. Cox proportional hazard models were carried out to determine the significance of variables associated with clinical outcomes. Log-minus-log plots was used to evaluate the proportional hazard assumption. All statistical significance were defined as *P* value < 0.05, which was based on two-sided tests.

## Results

### Patient characteristics

Table 1 lists the general clinical characteristics of 427 enrolled patients with 307 (71.9%) males and 120 (28.1%) females. The median age was 48 years (range: 17–82 years). There were 213 (49.9%) patients with locally advanced diseases (T3–4), 383 (89.7%) patients with nodal metastasis of the neck. In terms of tumor-node-metastasis (TNM) staging, there were 9 (2.1%) patients in stage I, 80 (18.7%) in stage II, 208 (48.7%) in stage III and 130 (30.5%) in stage IV. All patients completed the planned course of treatment with 59 (13.8%) patients who received radiotherapy alone and 368 (86.2%) patients who received concurrent chemoradiotherapy with or without neoadjuvant/adjuvant chemotherapy.

**Table 1:** Patient characteristics (*N* = 427)

Characteristics	*N*	%
Age (years)		
Median	48	
Range	17–82	
Sex		
Male	307	71.9
Female	120	28.1
Tumor classification^a^		
T1	77	18.0
T2	137	32.1
T3	134	31.4
T4	79	18.5
Nodal classification^a^		
N0	44	10.3
N1	152	35.6
N2	170	39.8
N3	61	14.3
TNM stage^a^		
I	9	2.1
II	80	18.7
III	208	48.7
IV	130	30.5
Treatment modality		
RT alone	59	13.8
CRT	368	86.2

*CRT* combined chemoradiotherapy, *RT* radiotherapy, *TNM* tumor-node-metastasis

^a^Tumor-node-metastasis staging system proposed by the American Joint Committee on Cancer (7th edition)

During a median follow-up of 67.5 months (range 4.8–85.5 months), there were 57 (13.3%) patients having local–regional recurrence, 64 (15.0%) patients experiencing distant metastasis, and 64 (15.0%) dead. The 5-year PFS and OS were 76.0% (median: 85.1 months, 95% CI 74.1–96.1 months) and 85.8% (mean: 77.8 months, 95% CI 76.0–79.6 months), respectively.

### The dynamic changes in hematological biomarkers

Table 2 presents the hematological biomarkers level at pre-treatment and post-treatment. Paired sample* t* test with Bonferroni correction revealed that ANC, APC, and ALC were significantly declined after therapy (*P* < 0.001 each), while NLR and PLR were elevated significantly (*P* < 0.001 each).

**Table 2:** Baseline hematologic markers at pre-treatment and post-treatment

Hematologic markers	Pre-treatment		Post-treatment		*P* value^a^
	Mean	SD	Mean	SD	
Neutrophil count, × 10^9^/L	4.14	1.51	3.55	1.46	< 0.001
Platelet count, × 10^9^/L	208.8	59.2	165.7	66.8	< 0.001
Lymphocyte count, × 10^9^/L	1.69	0.57	0.42	0.18	< 0.001
Neutrophil-lympocyte ratio	2.73	1.44	9.88	5.86	< 0.001
Platelet-lymphocyte ratio	135.33	61.44	447.55	229.31	< 0.001

*L* liter, *SD* standard deviation

^a^Paired *t* test with Bonferroni correction, *P* < 0.05

### The association of hematologic biomarkers at pre-treatment with clinical outcomes

Since the levels of hematological biomarkers at post-treatment were influenced by various factors, such as chemotherapy and nutritional support, therefore, we only analyzed the prognostic value of pre-treatment variables on survival.

**Fig. 1:**
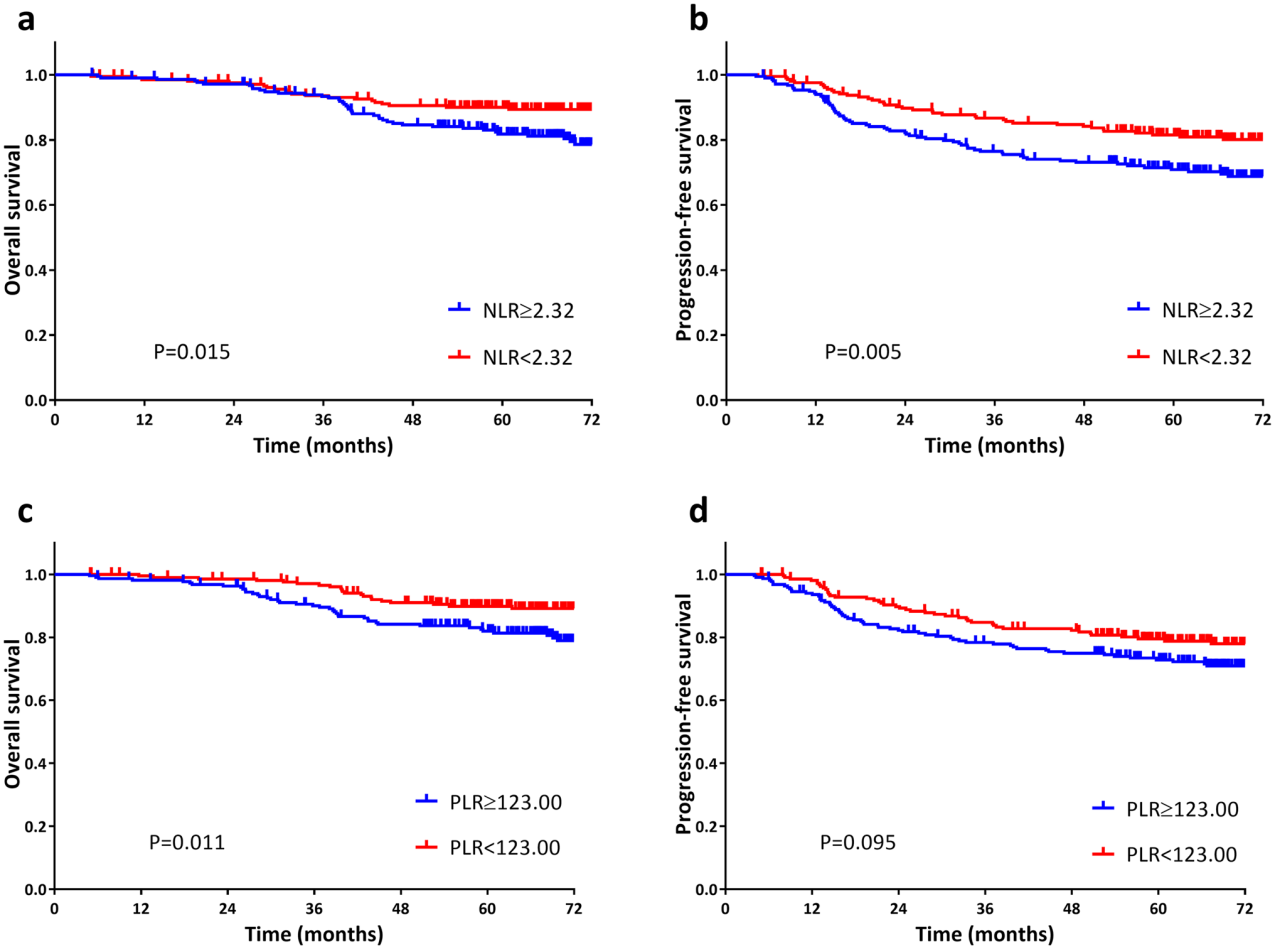
Kaplan-Meier survival curves of overall survival (**a** and **c**) and progression-free survival (**b** and **d**) according to pre-treatment neutrophil-lymphocyte ratio (NLR; **a** and **b**) and platelet-lymphocyte ratio (PLR; **c** and **d**). Log-rank test, *p* < 0.05

**Fig. 2:**
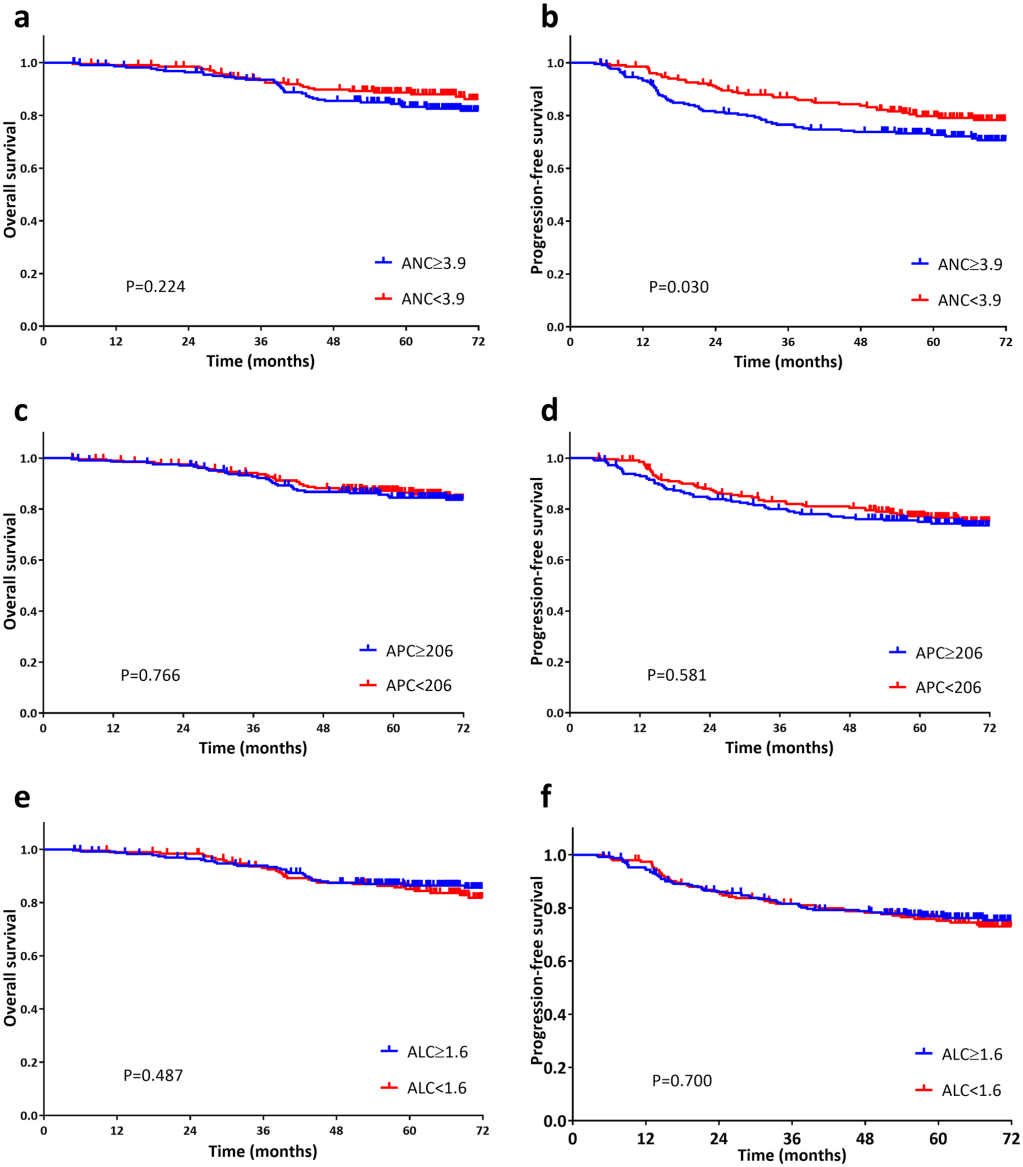
Kaplan-Meier survival curves of overall survival (**a**, **c** and **e**) and progression-free survival (**b**, **d** and **f**) according to pre-treatment neutrophil count (ANC; **a** and **b**), platelet count (APC; **c** and **d**) and lymphocyte count (ALC; **e** and **f**). Log-rank test, *p* < 0.05

Patients with NLR greater than or equal to 2.32 (NLR ≥ 2.32) were significantly inferior compared with those less than 2.32 in respect of 5-year OS (81.8 vs 90.0%, *P* = 0.015) and 5-year PFS (70.9 vs 81.5%, *P* = 0.005). The 5-year OS for those with PLR greater than or equal to 123.0 (PLR ≥ 123.0) was not reached for those whose PLR was less than 123.0 (81.9 vs 89.9%, *P* = 0.011). However, a significant difference of PLR subgroups was not observed with regard to 5-year PFS (72.8 vs 79.5%, *P* = 0.095; Fig. 1). Compared to patients with ANC lower than 3.9, those whose ANC greater than or equal to 3.9 (ANC ≥ 3.9) had worse 5-year PFS (72.7 vs 79.8%, *P* = 0.030), yet it was not associated with 5-year OS (83.2 vs 88.6%, *P* = 0.224; Fig. 2). APC subgroups (≥ 206 vs < 206) presented insignificant differences in either 5-year OS (84.5% vs 87.1%, *P* = 0.766) or PFS (74.9 vs 77.2%, *P* = 0.581). Furthermore, ALC subgroups (≥ 1.6 vs < 1.6) showed no obvious differences in OS (86.4 vs 85.1%, *P* = 0.487) or PFS (76.8 vs 75.2%, *P* = 0.700; Fig. 2).

Unadjusted Cox regression analysis showed that high NLR (≥ 2.32) and PLR (≥ 123.0) were significantly associated with inferior OS (*P* = 0.017, *P* = 0.012 each; Table 3); high ANC (≥ 3.9) and NLR (≥ 2.32) had significant reduction in terms of PFS (*P* = 0.032, *P* = 0.005 each; Table 4). Statistical differences were not observed in ANC, APC, or ALC subgroups with regard to OS (*P* = 0.225, *P* = 0.766, *P* = 0.488 each; Table 3), as well as APC, ALC, or PLR subgroups in PFS (*P* = 0.581, *P* = 0.700, *P* = 0.096 each; Table 4).

**Table 3:** Cox regression analysis for the association of pre-treatment hematological markers and overall survival

Variables	Unadjusted model		Adjusted model^a^	
	HR (95% CI)	*P* value	HR (95% CI)	*P* value
Neutrophil count				
< 3.9	Ref	0.225	Ref	0.281
≥ 3.9	1.361 (0.827–2.242)		1.316 (0.798–2.171)	
Platelet count				
< 206	Ref	0.766	Ref	0.468
≥ 206	1.077 (0.660–1.759)		1.201 (0.732–1.972)	
Lymphocyte count				
< 1.6	Ref	0.488	Ref	0.953
≥ 1.6	0.841 (0.515–1.373)		0.985 (0.597–1.625)	
NLR				
< 2.32	Ref	**0.017**	Ref	**0.048**
≥ 2.32	1.872 (1.118–3.137)		1.699 (1.005–2.873)	
PLR				
< 123.00	Ref	**0.012**	Ref	**0.032**
≥ 123.00	1.933 (1.154–3.239)		1.765 (1.051–2.964)	

*CI* confidence interval, *HR* hazard ratio, *NLR* neutrophil–lymphocyte ratio, *PLR* platelet-lymphocyte ratio, *Ref* reference

*P* values < 0.05 are in bold

^a^Adjusted for age, gender, TNM stage and treatment modality

**Table 4:** Cox regression analysis for the association of pre-treatment hematological markers and progression-free survival

Variables	Unadjusted model		Adjusted model^a^	
	HR (95% CI)	*P* value	HR (95% CI)	*P* value
Neutrophil count				
< 3.9	Ref	**0.032**	Ref	0.056
≥ 3.9	1.530 (1.038–2.256)		1.461 (0.990–2.155)	
Platelet count				
< 206	Ref	0.581	Ref	0.480
≥ 206	1.113 (0.761–1.626)		1.148 (0.783–1.683)	
Lymphocyte count				
< 1.6	Ref	0.700	Ref	0.923
≥ 1.6	0.928 (0.635–1.356)		0.981 (0.665–1.448)	
NLR				
< 2.32	Ref	**0.005**	Ref	**0.008**
≥ 2.32	1.747 (1.181–2.585)		1.710 (1.150–2.543)	
PLR				
< 123.00	Ref	0.096	Ref	0.161
≥ 123.00	1.385 (0.943–2.033)		1.318 (0.896–1.939)	

*CI* confidence interval, *HR* hazard ratio, *NLR* neutrophil–lymphocyte ratio, *PLR* platelet-lymphocyte ratio, *Ref* reference

*P* values < 0.05 are in bold

^a^Adjusted for age, gender, TNM stage and treatment modality

After adjustment for some potential confounders, including age, TNM staging, and treatment modality, high NLR (≥ 2.32) was still significantly inferior in OS (HR 1.699, 95% CI 1.005–2.873, *P* = 0.048; Table 3) and PFS (HR 1.710, 95% CI 1.150–2.543, *P* = 0.008; Table 4). Moreover, high PLR (≥ 123.0) remained significantly related to worse OS (HR 1.765, 95% CI 1.051–2.964, *P* = 0.032; Table 3), yet it was not correlated with PFS (HR 1.318, 95% CI 0.896–1.939, *P* = 0.161; Table 4). Although high ANC (≥ 3.9) was insignificant in case of PFS (HR 1.461, 95% CI 0.990–2.155, *P* = 0.056; Table 4), its clinical value remained worth noting.

### Discussion

As a highly radio-sensitive disease, radiotherapy remains the first-line treatment for non-disseminated NPC. The development of IMRT is a breakthrough in the treatment of NPC. Compared with two-dimensional radiotherapy (2D-RT) and three-dimensional conformal radiotherapy (3D-CRT), IMRT generates higher radiation dosage to tumor volume with better target coverage and normal tissue sparing. IMRT has been proven with ideal local control in NPC [10], nevertheless, distant control remains inadequate. Individual therapy selection should be established for optimal prognosis. Besides treatment techniques, the biological variability of tumors cannot be overlooked any more.

Clear differences were found between levels of hematological markers tested at pre- and post-treatment in our present study. ANC, APC and ALC level showed significant decrease, while NLR and PLR increased significantly at post-treatment. Decreased ANC, APC and ALC level were most likely due to malnutrition as an impact of poor oral intake and increased protein catabolism [11, 12]. Both clinical conditions can lead into subsequent immunosuppression. On the other side, increased NLR and PLR were most likely because of systemic inflammation and critical lymphopenia caused by the treatment administered.

The relationship between inflammation and cancer has been reported to be interactive and synergetic. The sites of chronic inflammation often have greater risk of neoplasia [13, 14]. Elevated level of inflammatory cells and cytokines infiltration were frequently present in tumor biopsies [13]. Inflammatory factors in situ facilitate angiogenesis of tumor, inhibition of adaptive anti-tumor immunity, as well as elicit insensitivity of hormones regulation. Moreover, neoplasms further release cytokines and chemokines into the systemic circulation to regulate the level of lymphocytes, neutrophils and platelets [15, 16].

Lymphocytes, as important components of immune surveillance, induce tumor rejection by specific recognition of tumor-related antigens (TAs) [17]. It has been reported that a higher occurrence of neoplasms was observed in mice with a defect of lymphocytes [18], or some key components of T cell effectors, such as perforin [19], interferon-γ (IFN-γ) [20]. Further study revealed tumor regression due to intensified response of T lymphocytes, implemented through administration of interleukin 2 (IL-2) or autograft of tumor-infiltrating lymphocytes (TILs) [21, 22]. Nowadays, immune checkpoint inhibitors have achieved notable success in a variety of cancers, which potentiate lymphocyte responses by specifically effecting on cytotoxic T lymphocyte-associated antigen 4 (CTLA-4) or programmed cell death protein 1 (PD-1) [23].

Neutrophils are major indicators of inflammation and infection. It is modulated by various cytokines, of which, interleukin-8 (IL-8) [24], macrophage inflammatory protein-1β (MIP-1β) [25], as well as macrophage migration inhibitory factor (MIF) [26] have been widely studied to implicate in development and progression of tumors. Meanwhile, neutrophils can further produce kinds of cytokines to facilitate carcinogenesis [27]. The leukemoid reaction in NPC was regarded as the primary manifestation of malignancy or relapse [28]. Peripheral neutrophils from HNSCC patients presented with a longer life span [7]. The long-lived and activated neutrophils contributed to tumor cell metastasis [29]. Peng et al. [30] further verified that tumor cells were less available to migrate with inhibited neutrophil infiltration, and the ability restored by re-infiltration. Furthermore, in vitro study reported that the responses of cytotoxic T lymphocytes were inhibited by infiltrating neutrophils, which effect was directly proportional to the amount of neutrophils [31].

Platelet, a regulatory factor in thrombosis and hemostasis, contributes a lot in tumor growth, extravasation and dissemination [32]. It is well acknowledged that hypercoagulable state is frequently presented in cancer patients [33]. The activated platelets shield tumor cells from immune eliminations, and escort them to distant sites driven by platelet-derived transforming growth factors (TGF-β) [34]. Labelle et al. [34] also demonstrated that platelet inactivation with ablation of nuclear factor kappa B (NF-κB) pathways in tumor cells or TGF-β1 could suppress the metastatic potential of tumor cells. Other study [35] revealed that platelet-derived CXCL5 and CXCL7 chemokines sped up the development of prometastatic microenvironments. Moreover, either blockade of CXCL5/7 receptor CXCR2 or depletion of platelets would slow down the metastatic process.

Our study presented NLR and PLR were independent prognosticators for survival in NPC patients receiving IMRT-based therapy. High NLR was significantly associated with inferior OS and PFS, and high PLR was correlated with poor OS. Yet, ANC, APC or ANC were insignificant. There has been a mass of studies elaborating the significant relation of ANC, APC, ALC, NLR or PLR with prognosis in different types of cancers, as well as HNSCC [1, 2, 8, 9]. However, the cutoff values were discordant. Su et al. [1] illustrated that initial ANC > 8 × 10^9^/L was correlated with poor OS, PFS and DMFS, and APC > 10 × 10^9^/L indicated inferior OS and PFS in NPC patients. He et al. [2] once divided initial peripheral lymphocytes, neutrophil and NLR from NPC patients into quarters, patients with highest either lymphocyte percentage or ALC had superior PFS compared with those of lowest quartiles. While highest neutrophil percentage or NLR > 2.74 predicted poor PFS. Sun et al. [9] reported that NPC patients with NLR ≥ 2.7 or PLR ≥ 167.2 indicated shorter PFS, while PLR ≥ 163.4 was correlated with poor OS. Lu A et al. [36] revealed that NLR ≥ 2.28 indicated poor OS and PFS, with PLR ≥ 174 correlated with inferior OS. Lin et al. [37] believed the combination of platelet with NLR (COP-NLR score) was a better predictor compared with NLR alone or APC alone. A latest meta-analysis by Su et al. [38] evaluated the hematological parameters in NPC patients, and suggested the significant role of NLR and lymphocyte in prognostic prediction. As a matter of fact, it is hard to pinpoint a unified value since each cancer has its own specific bio-physiological characteristics. In addition, the baseline levels of biomarkers were diverse. On the other hand, even for the same type of cancer, the differences in measurement exist between different instruments.

There were several advantages in our study. Though the relationship of hematological biomarkers with survival has been studied profoundly in HNSSC, we specifically focused on NPC. Since IMRT technique has been the mainstream, it is noteworthy that we carried out this study with both large-population sample size and IMRT-based therapeutic approach. Moreover, we took multiple hematological variables into consideration.

The inadequacy of our study was the lack of other inflammatory indicators, such as C-reactive protein (CRP), erythrocyte sedimentation rate (ESR) and lactate dehydrogenase (LDH) for more comprehensive analysis. Further randomized trials of large-scale, multi-center studies are needed to elucidate the clinical values of hematological markers on prognosis and therapy plan decision.

## Conclusion

In conclusion, high NLR level was significantly associated with inferior OS and PFS, while high PLR level correlated with poor OS. Furthermore, high ANC level presented with a tendency towards shorter PFS, even though its statistical test was insignificant, it may be clinically valuable with the expansion of population. Due to its easily accessible and economically feasible characteristics, hematological biomarkers may be used in clinical setting to predict NPC mortality.
